# What do we actually want to experience? A computational metric for assessing reward values

**DOI:** 10.21203/rs.3.rs-5875678/v1

**Published:** 2025-02-17

**Authors:** Christian E. Waugh, Adam P. Porth, Xuanyu Fang, L. Paul Sands, Kenneth T. Kishida

**Affiliations:** 1 Department of Psychology, Wake Forest University, 1834 Wake Forest Rd. Winston Salem, NC USA.; 2 Department of Physiology and Pharmacology, Wake Forest School of Medicine, Winston Salem, NC USA; 3 Department of Neurosurgery, Wake Forest School of Medicine, Winston Salem, NC USA

**Keywords:** reward, motivation, computational, reinforcement learning

## Abstract

People’s motivation to have different experiences is predicated on how much they find those experiences rewarding or not, and these reward values are not always fully accessible to our consciousness. In two studies, we demonstrate that using a combination of reinforcement learning (RL) paradigms and computational modeling, we can measure computationally inferred reward values (cRV) of experiences, which do not rely on conscious self-report. Consistent with motivational reward theory, convenience samples of participants exhibited higher cRV (greater reward value of that experience) to viewing positive vs. negative images (subject pool; Study 1) and to viewing more vs. less attractive faces (online sample; Study 2). Further, these cRVs were sensitive to context (familiarity vs. novelty of images, Study 1) and to individual differences (attraction preference, Study 2). Lastly, although cRVs were mildly correlated with explicit self-report values, which demonstrates their validity, they were better predictors of behavior than were the explicit values, which suggests that cRVs are capturing reward processes that are not represented by explicit value judgments. This method of measuring cRV holds great promise for understanding the motivation driving people’s choices of a variety of experiences across a wide array of fields of study.

People differ in their choices to have an experience, and these motivated behaviors are predicated on how they evaluate the potential reward and punishment values of these experiences (Berridge, 2018). Indeed, findings from early behavioral research (Skinner, 1938; Thorndike, 1933), modern neuroscience (McClure et al., 2003; Schultz et al., 1997), and clinical research (Sherdell et al., 2012; Treadway et al., 2012) support the idea that understanding how humans assign value to stimuli and experiences is central to explaining behavior. Therefore, accurately measuring how people value different experiences as targets for motivated behavior, both consciously and unconsciously, is critical for advancing our understanding of human psychology and behavior (Berridge, 2018; Berridge & Robinson, 2003; Berridge & Winkielman, 2003).

The dominant method in the literature to measure these reward values in humans is to acquire some consciously accessible self-report of reward value (Bartra et al., 2013). Although these self-report metrics can be fair predictors of motivated behavior, it is clear that they fail to account for other aspects of reward value that may be less consciously accessible but still powerful predictors of behavior (Anselme & Robinson, 2016). These conscious self-reports of reward value tend to be associated with consciously accessible goals and attitudes (‘liking’, Berridge & Robinson, 2003) and less related to those goals and attitudes that are not as consciously accessible (such as those that drive some behaviors in drug addiction, Fischman & Foltin, 1992). An evolutionarily ancient system, reward/punishment motivation is supported primarily by subcortical neural circuitry (Berridge & Robinson, 2003; Mobbs et al., 2020), which can drive behavior regardless of whether one eventually becomes consciously aware of the associated reward value or not (Berridge, 2018). For example, researchers found that subliminally priming participants with emotional expressions impacted their enjoyment of novel drinks but not their conscious feelings (Winkielman et al., 2005).

It is clear, then that to fully understand motivated human behavior, researchers must also use indirect metrics of reward value that do not rely on explicit self-report. Some metrics have been developed that are promising, but, we argue, do not fully capture a scalar metric for non-self-reported reward value. Implicit association tests (IATs, Greenwald et al., 1998) and affect misattribution tests (AMP; Payne & Lundberg, 2014) measure people’s attitudes or ‘liking’ of certain stimuli/ideas, but although the liking system informs the motivational ‘wanting’ system (Berridge & Robinson, 2003), they rely on different mechanisms and can be dissociated (Salamone et al., 1997; Sherdell et al., 2012; Treadway et al., 2012; Waugh & Gotlib, 2008). On the other hand, approach-avoidance tests (Krieglmeyer & Deutsch, 2010) measure motivational reward value, but only for highly appetitive and aversive stimuli that engender strong musculature-based approach-avoidance behaviors (Krieglmeyer & Deutsch, 2010).

We propose that reinforcement learning (RL) paradigms and the computational approaches to assess RL processes can be used as an indirect measure of reward value that are not based on self-report (McClure et al., 2003). In RL, people make a series of choices between actions (e.g., selecting a cue) that correspond to different probabilities of experiencing some positive and/or negative outcome (reward or punishment, Sutton & Barto, 2018). Typically, the outcome of the choice is presumed and input into the RL model. For example, in a monetary experiment, the assumed reward value of winning $5 is presumed to be 5 times greater than the presumed reward value of winning $1 and presumed to be the same reward value for everyone.

In addition to the problems with these assumptions, not all experiences have direct quantitative values that one can assume maps onto reward value. There is no inherent quantitative reward value of eating strawberries or toast that one can assume without assessing it for each person separately. Katahira et al. (2015) developed a computational method of estimating the reward value of choice outcomes instead of assuming them. In this method, which we term kRL (reward value will be measured by κ parameters in the model), participants make choices that lead to different experiences (e.g., viewing emotional images) and their pattern of choices can be computationally modeled to infer their reward values of these experiences (Katahira et al., 2015). We have coined these measurements *computationally inferred reward values* (cRV) to reflect that these are not explicit self-reported values but computationally inferred from choice behavior and to distinguish these from the reward values typically assigned in traditional RL studies. With this kRL method, there are no strong assumptions about the quantitative value of these cRVs, which allows for variation across people, and for these cRVs to change because of drive state (e.g., hungry, anxious) or context.

In two studies, we tested this kRL method of assessing the cRVs of viewing emotional images (Study 1) and faces that vary in their attractiveness (Study 2) to examine if these cRVs are sensitive to context and/or individual differences and to examine whether these cRVs are correlated with explicit self-report reward values.

## Study 1

In Study 1, we aimed to demonstrate that the Katahira et al., (2015) kRL method of assessing cRVs toward viewing positive and negative emotional imagery is replicable and can form the foundation for understanding the psychological underpinnings of reward value. Given that positively and negatively valenced stimuli tend to map onto reward and punishment motivation (Lang et al., 1990), we hypothesized that participants’ cRVs of viewing positive images will be greater than that for viewing negative images (Katahira et al., 2015). Participants viewed some of the emotional images before the choice task, which allowed us to explore whether this kRL method is sensitive enough to pick up on possible changes in cRVs to familiar (vs. novel) emotional images due to processes such as habituation (Dijksterhuis & Smith, 2002), sensitization (Bradley et al., 1996), or exposure (Zajonc, 2001). Lastly, participants provided explicit self-report ratings of the emotional valence of some of these images, which allowed us to explore whether the cRVs correlate with the explicit values of these stimuli.

### Method

#### Participants

One hundred and fifty-four participants (_95_ Females, 59 Males; M_age_ = 18.59, SD_age_ = .75) were recruited from the Introductory Psychology subject pool at Wake Forest University. Ethnic/racial representation was as follows: 68.8% White/Caucasian, 23.4% Asian, 3.9% Black/African-American, 0.6% American Indian or Alaskan Native, 1.2% identified with more than one race/ethnicity, and 1.9% unknown/not reported. In addition, 8.4% of participants identified as Hispanic/Latino. To take part in the study, participants had to be over 18 years old or 17 years old with the permission of a parent. Participants’ data were excluded if they had more than 30 missing choices for a given trial type (n = 12), chose only one side of the screen (n = 1), and/or only chose one fractal for a given trial type (n = 4), resulting in a final N = 137.

#### Sample size justification

Our target sample size was 150, which was based on prior RL studies that index individual differences (*n* = 151; Heller et al., 2018), and is significantly more than the study that we were attempting to replicate (*n* = 30; Katahira et al., 2015).

#### Materials

We used fractal images from online sources to represent choices because they are neutral cues with which the participants have no prior experience.

To have enough emotional pictures in the pool to account for participant’s choices, we compiled two sets of emotional pictures each with 68 positive, 40 neutral, and 68 negative pictures. Picture information can be found in Supplementary Online Material.

#### Emotion rating task

Both of the tasks were built for online execution with PsychoPy (Peirce et al., 2019). Participants first viewed one of the two picture sets (“familiar”) and rated the emotion they felt toward each picture on a 1 (most negative) to 9 (most positive) scale.

#### Emotion choice task

The second task was the emotion-choice task, which comprised 160 decision trials divided into two trial types; there were 80 decision trials with the familiar pictures (those they had viewed and rated in the prior emotion rating task), and 80 decision trials with the novel pictures (those they were seeing for the first time). Familiar and novel decision trials were randomized.

In the decision trials, participants were shown two fractal images, chosen randomly at the beginning of the task from a pool of fractals. The fractals served as visual cues representing two possible choices. The fractal images for the familiar and novel trials were different sets, so the cRVs for the two trial types could be calculated separately.

For each decision trial, one fractal image was designated to be an “advantageous” choice (65% positive, 20% neutral, 15% negative), and the other fractal was designated to be the “disadvantageous” choice (15% positive, 20% neutral, 65% negative). The advantageous and disadvantageous probabilities switched between the two fractal images at predetermined times for both trial types to prevent a learning plateau. The side of the screen occupied by each fractal image was randomly switched among trials so that the participants’ learning was linked to the fractals instead of the side of the screen. Participants’ were more likely (than chance: .5) to choose the advantageous option for both familiar (M = .55, CI_95_ [.53, .56], *t*(136) = 5.33, p < .001, and novel trials (M = .54, CI_95_ [.52, .56]), *t*(136) = 4.14, p < .001.

**Figure 1.**
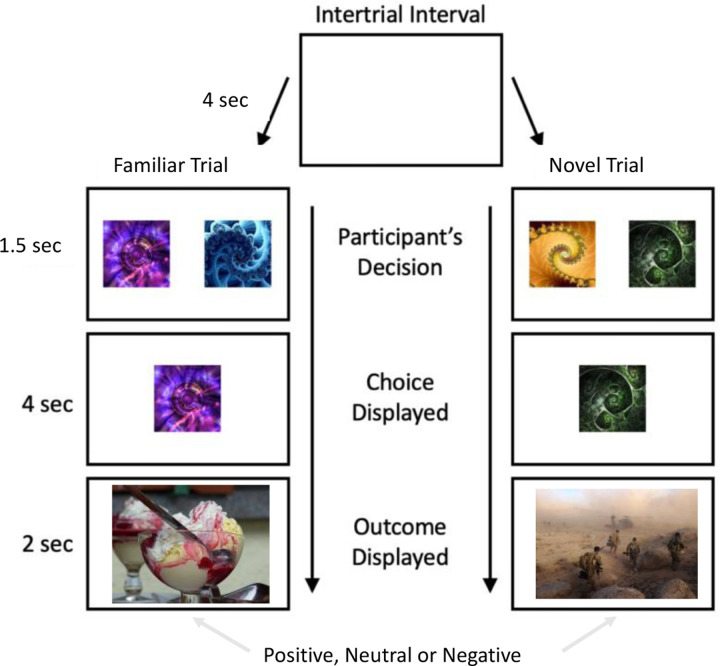
Study 1 task structure.

Participants were given 1.5 seconds to make their decision between the two fractals. After their decision, the chosen fractal was displayed for four seconds, and the resulting picture was presented for two seconds. Between each trial, there was a four-second intertrial interval. An illustrative example of the decision-making task procedure is shown in Figure 1.

#### Procedure

After signing up for the study through the subject pool system, participants had 5 days to complete the study online on their own computers at a time convenient to them. After providing informed consent, participants provided demographic information and were then redirected to Pavlovia.org, an online experiment platform, where they completed the emotion ratings task, and then the emotion choice task. Importantly, the instructions for the emotion choice task just told the participants to make a choice and that they would see their choice and then a picture afterwards. They were not told about the contingencies between their choices and the pictures, nor motivated to see the “best” pictures. The study took approximately 60 minutes to complete. All procedures were approved by the Institutional Review Board at Wake Forest University.

#### Analysis

##### Q-Learning kRL Model.

To assess cRV we used Q-Learning to model participants’ choices separately for the familiar and novel trial types (Sutton & Barto, 2018). The standard Q-learning model calculates *Q*, which is the expected value of making a particular choice (which, in our case was tied to specific fractals). This *Q* value is updated after each choice given the outcome of that choice using the following equations:

$$Q_{i}(t+1)=Q_{i}(t)+\alpha \cdot \delta (t),$$

$$\delta (t)=\nu (t)-Q_{i}(t)$$

*Q*_*i*_(*t*) is the *Q* value for option *i* (which corresponds to a certain fractal image presented to the participants) on trial *t*. The *Q* value is updated on each trial by summing the current *Q* value for option *i* with the product of the learning rate (*α*; where 0 ≤ *α* ≤ 1) and the reward-prediction error (*δ*) for trial *t*. The learning rate (*α*) represents the strength with which *Q* values are updated on each trial. The reward-prediction error represents the differences in received and predicted rewards. It is calculated using the reward value (*v*) for trial *t* subtracted by the current expected value, *Q*_*i*_(*t*). In most models, this reward value *r(t)* is inputted into the equations as some quantitative outcome of that choice (e.g., money won or lost on that trial). We, instead, adapted Katahira (2015) in parameterizing *v* so that we can calculate cRV as demonstrated below:

For the *t*-th trial of the familiar trials:

$$\nu (t)=\{\begin{array}{ll} \kappa_{\text{fam}}^{\text{Pos}} & \text{if}\;\text{a}\;\text{positive}\;\text{picture}\;\text{was}\;\text{presented}\\ 0 & \text{if}\;\text{a}\;\text{neutral}\;\text{picture}\;\text{was}\;\text{presented}\\ \kappa_{\text{fam}}^{\text{Neg}} & \text{if}\;\text{a}\;\text{negative}\;\text{picture}\;\text{was}\;\text{presented} \end{array}$$

For the *t*-th trial of the novel trials:

$$\nu (t)=\{\begin{array}{ll} \kappa_{\text{nov}}^{\text{Pos}} & \text{if}\;\text{a}\;\text{positive}\;\text{picture}\;\text{was}\;\text{presented}\\ 0 & \text{if}\;\text{a}\;\text{neutral}\;\text{picture}\;\text{was}\;\text{presented}\\ \kappa_{\text{nov}}^{\text{Neg}} & \text{if}\;\text{a}\;\text{negative}\;\text{picture}\;\text{was}\;\text{presented} \end{array}$$

The *κ*^Pos^ and *κ*^Neg^ parameters quantify cRV for positive and negative pictures, respectively. Instead of also dynamically parameterizing the reward value of neutral pictures throughout the decision-making task, we followed Katahira et al. (2015) in setting their *v* as 0 and parameterizing the initial *Q* value: *Q*_init_. By doing so, *Q*_init_ reflects the starting estimated reward value for the neutral pictures and serves as a referent for *κ*^Pos^ and *κ*^Neg^ such that positive and negative cRVs can be understood as reflecting values that are either higher or lower than *Q*_init_, respectively. The *Q*_init_ is less interpretable by itself and will not be reported.

To model choices, we implemented the soft-max policy function, which represents the probability of choosing one of two options given the Q values of each option, and a temperature parameter (*β*) that reflects the degree to which participants explored their options (high beta) or exploited the higher Q value option (lower beta):

$$P[a(t)=1]=\frac{\text{exp[}\beta *Q_{1}\text{(t)]}}{\text{exp[}\beta *Q_{1}\text{(t)]}+\text{exp[}\beta *Q_{2}\text{(t)]}}$$

##### Hierarchical Bayesian Analysis Using the Stan Package.

We used Stan language (Stan Development Team, 2022) in conjunction with R (hBayesDM; Ahn et al., 2017) to conduct Hierarchical Bayesian Analysis (HBA; Ahn et al., 2011) to assess parameters for each participant. HBA allows for the estimation of both group and individual parameters simultaneously in a mutually constraining fashion (i.e., able to include both individual differences and group tendencies). Prior to the full HBA estimation, we set ranges for parameter values from a combination of conventions (*α*: .1 to 1.0, *β*: 0 to 20; Ahn et al., 2017; Sands et al., 2023) and prior ranges of these values (*κ*^Pos,Neg^ : − 1.5 to 1.5, *Q*init: − 1.5 to 1.5; Katahira et al., 2015) and then used Stan’s variational algorithm to approximate initial values. Next, we approximated a posterior distribution of parameter estimations for both familiar and novel trial types and extracted the median parameters of each participant to input into subsequent statistics (for full reporting on parameters see Table S1). To confirm proper model fitting, we inspected the convergence of the Markov chains of the Stan language’s Hamiltonian Monte Carlo sampler by verifying sufficient chain mixing visually and via the Gelman-Rubin statistic R-hat (i.e., the ratio of within- and between-chain variance), which was less than 1.1 for almost all parameters across all models.

We report estimated marginal means and standard errors for the t-tests as well as 95% confidence intervals, and effect sizes (d).

### Model testing

To discern whether the cRV model (*α*, *β*, *κ*^Pos,Neg^, *Q*_init_) was a better fit to the behavioral data than simpler, alternative models we used leave-one-out cross-validation, which can estimate out-of-sample prediction accuracy from fitted Bayesian models (Vehtari et al., 2017). We used the familiar trials so that we could also include a model with the explicit ratings.

#### Model α.

We just estimated the *α* parameter. This model did not have a temperature adjustment (*β*) in the softmax equation and used 1, 0, and −1 to represent reward values from viewing positive, neutral and negative images, respectively.

#### Model αβ.

We added the temperature (*β*) parameter adjustment to the softmax equation from Model ***α***.

#### Model αβExp.

In addition to the *α* and *β* parameters, we imported the explicit ratings from the ratings task (for familiar trials) to represent the reward values in the decision-making task. Before estimation, we standardized each participant’s ratings.

#### Model αβκ.

The cRV model in which we estimated *α*, *β*, *κ*^Pos,Neg^, *Q*_init_, as described above.

**Figure 2.**
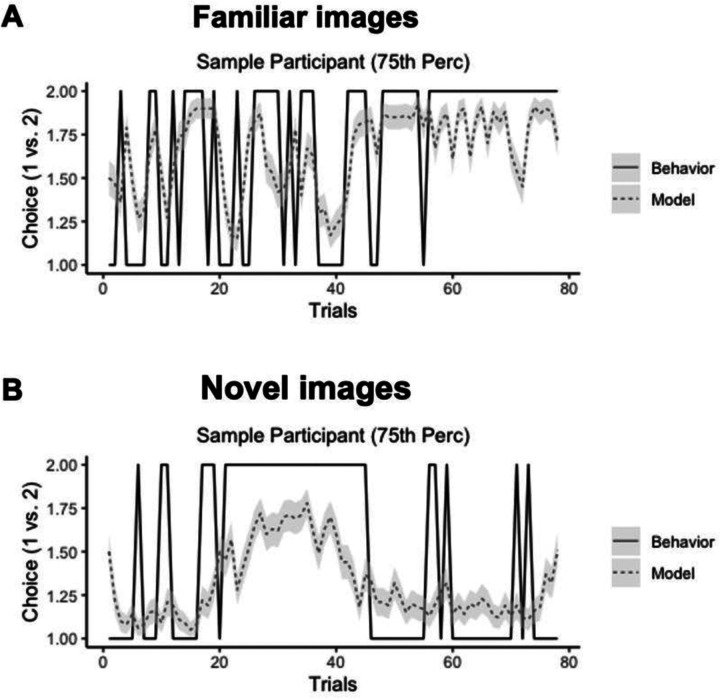
Study 1: Sample participant (75^th^ %ile) with model predicted accuracy (average over 100 iterations) and actual choice during the task **(A:** familiar image trials, **B:** novel image trials).

As evidenced in Table 1, the cRV model ***αβκ*** had the best prediction accuracy of the models we tested, thus justifying its use in our study and demonstrating that these computationally inferred reward values offered information above and beyond that provided by participants’ explicit choices (model ***αβExp***). To illustrate this prediction accuracy of the cRV model, we calculated the predicted choices for each trial given participants’ estimated model parameters (in 100 iterations) and showed that these models predicted choice accuracy greater than chance (Familiar: *M* = .59, Wilcoxon’s test = 8874.5, *p* < .001; Novel: *M* = .58, Wilcoxon’s test = 8152.5, *p* < .001). We illustrate these models with sample participants whose models accurately predicted their behavior at the 75^th^ percentile of the sample (Figure 2ab).

### Exploration of model with fewer trials

Given that reward values can change throughout the task due to satiation, desensitization and/or habituation processes (Bradley et al., 1996; Dijksterhuis & Smith, 2002; Rolls, 2015) we also explored whether modeling the first 40 trials instead of all 80 altered any cRV patterns. We report modeling parameters and statistics for the 40-trial version in Supplementary Online Material and note any differences in the reward patterns in the results section. Notably, the 40-trial version exhibited a similarly good fit to the data, warranting its exploration here.

### Transparency and Openness

Study 1 was not preregistered; the preregistration for Study 2 can be accessed at https://osf.io/tak27. Deidentified data for both studies along with the data-analysis scripts are posted at https://osf.io/85wx3/. Researchers interested in assessing cRV for their own purposes can find a template for running this task and analyzing its data at https://gitlab.pavlovia.org/WakeEmoLab/rlposnegtemplate/.The materials used in these studies are widely available.

### Results

As expected, participants explicitly rated the positive pictures (*M* = 6.53, CI_95_ [6.25, 6.81]) significantly higher than the neutral pictures (*M* = 4.08, CI_95_ [3.91, 4.25]), *t*(136) = 16.82, *p* <.001, which were rated significantly higher than the negative pictures (*M* = 2.50, CI_95_ [2.26, 2.74]; Figure 2), *t*(136) = 11.65, *p* <.001. There were no significant rating differences between the positive and neutral picture sets that were counterbalanced among participants, ts < .87, ps > .383, however, one of the negative picture sets was slightly more negative than the other, (M_1_ = 2.78, M_2_ = 2.23), *t*(135) = 2.35, *p* =.020, CI_95_ [.09, 1.02]. This supports our use of counterbalancing to account for this unforeseen difference in picture sets.

**Figure 3.**
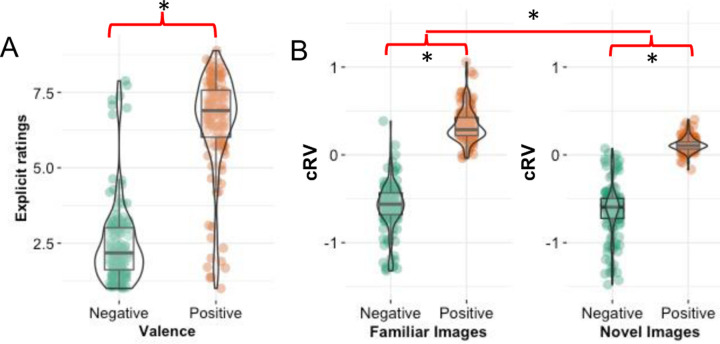
Study 1: Explicit ratings for familiar positive vs. negative images (A) and computationally inferred reward value (cRV) differences (B) for familiar vs. novel positive and negative images. * p < .05

Next, to test the hypothesis that cRV reflects reward differences between positive and negative emotional stimuli and to explore whether familiarity moderates these cRV differences, we conducted a 2 (Valence: positive, negative) × 2 (Familiarity: familiar, novel) ANOVA on the cRV parameters (κ values) and explored interactions with paired t-tests. As hypothesized there was a strong main effect of Valence, *F*(1,136) = 828.96, *p* <.001, η_p_^2^ = .86, with higher cRV for positive pictures (*M* = .22, CI_95_ [.20, .24]) than for negative pictures (*M* = −.63, CI_95_ [−.68, −.58]; Figure 3). There was also a main effect of Familiarity, *F*(1,136) = 92.25, *p* <.001, η_p_^2^ = .40, but both of these were qualified by an interaction of Valence and Familiarity, *F*(1,136) = 34.90, *p* <.001, η_p_^2^ = .20. Decomposing the interaction shows that although the cRV for familiar pictures was significantly greater than the cRV for novel pictures for both positive and negative pictures, this difference was much greater for the positive pictures (*M*_*familiar*_ = .33, CI_95_ [.30, .37]; *M*_*novel*_ = .11, CI_95_ [.10, .13]), *t*(136) = 14.64, *p* <.001, CI_95_ [.19, .25], d = 1.25, than for the negative pictures (*M*_*familiar*_ = −.60, CI_95_ [−.65, −.55]; *M*_*novel*_ = −.65, CI_95_ [−.71, −.60]), *t*(136) = 2.23 *p* =.028, CI_95_ [.01, .10], d = .19. These patterns were the same for the 40-trial version (Supplementary Online Material).

**Figure 4.**
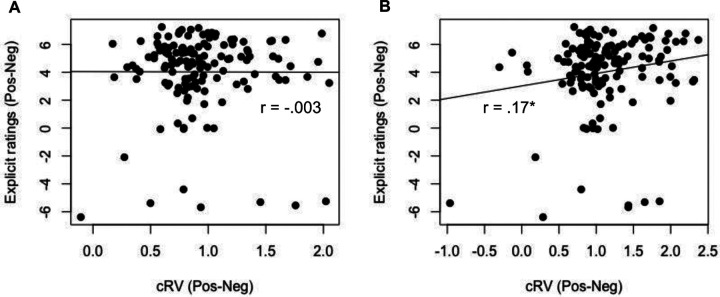
Correlation between explicit ratings and computationally inferred reward values (cRV) for familiar images for the 80-trial version (A) and 40-trial version (B). * p < .05.

Lastly, we explored the relationship between the explicit ratings and the cRVs within each valence. There was not a significant correlation between the explicit ratings and cRVs for positive, *r*(136) = −.003, *p* =.976, nor negative pictures, *r*(136) = −.08, *p* =.326. Because the cRVs are more interpretable when contrasted with each other, we also computed cRV (*κ*^Pos^ - *κ*^Neg^) and explicit rating (positive images – negative images) difference scores and correlated these with each other. The explicit rating and cRV difference scores were also not significantly correlated, *r*(136) = −.003, *p* =.968 (Figure 4a).

Notably, these patterns were different for the 40-trial version. There was a significant correlation between explicit ratings and cRVs for positive pictures, r(136) = .17, p = .043, (but not negative pictures again, r(136) = .07, p = .40) and a mild, but significant positive correlation between explicit rating and cRV difference scores, r(136) = .17, p = .049 (Figure 4b).

### Discussion

In Study 1, we replicated Katahira (2015) that this kRL method can capture that the cRVs of viewing positive images is higher than that of viewing negative images, which supports the affective mapping of valence onto reward/punishment motivations (Lang et al., 1990). Furthermore, the cRVs of viewing familiar positive images was higher than that for viewing novel positive images, which suggests that we successfully captured the effects of mere exposure on these images – the process by which the positivity of non-aversive stimuli increases with greater exposure (Zajonc, 2001). Lastly, there was preliminary evidence that this method captured reward values that provide additional information above and beyond explicit self-reported reward values. There was a positive correlation between cRVs and explicit values (for the 40-trial version), which validates cRVs as indicators of the rewarding properties of valenced emotional images. However, this was a mild correlation and allowing the model to estimate the cRVs led to better predictive accuracy than using the participants’ explicit values as reward values. These findings suggest that cRVs are capturing reward processes that are at least partially separable from those represented by these explicit self-reported values.

## Study 2

In Study 2, we assessed whether the kRL method can also capture the reward value of social rewards such as viewing attractive faces. Based on theories of beauty (Etcoff, 2011) and studies measuring gaze preferences for (Langlois et al., 1987) and neural responses in reward regions to attractive faces (Aharon et al., 2001), we hypothesized that the cRVs for viewing attractive faces would be higher than that for viewing less attractive faces. We recruited participants who were either primarily attracted to males or to females to explore whether the kRL method can also track potential attraction-consistent individual differences in cRV (Chivers, 2017). Lastly, participants rated the attractiveness of the faces to provide an explicit value judgment to again test the relationship between cRVs and explicit reward values as in Study 1.

### Methods

#### Participants

One hundred seventy-five participants were recruited from Prolific, an online recruiting platform for scientific studies (www.prolific.co). Based on preregistration exclusion criteria, all participants were screened to ensure they were primarily attracted to either males or females (androphilic or gynephilic) and from the United States. There were 98 gynephilic (primarily attracted to females; 95 heterosexual males and 3 homosexual females) and 77 androphilic (primarily attracted to males; 64 heterosexual females and 13 homosexual males) participants, with their ages ranging from 18 to 64 (*M* = 38.91, *SD* = 12.17). We included homosexual participants in our study as they share similar attraction patterns as heterosexual participants. One thirty-seven participants self-identified as White/Caucasian (78.3%), 18 self-identified as Black/African American (10.3%), 11 self-identified as Asian (6.3%), 1 self-identified as American Indian or Alaskan Native (0.6%), 1 self-identified as other (0.6%; South Asian), and 7 self-identified as multiracial (4.0%); 8 self-identified as Hispanic/Latino (4.6%). The data from 39 participants were excluded based on preregistration criteria. Reasons for data exclusion include only choosing one decision option per gender (27 participants), having more than 30 missing data points from one gender (1 participant), and not having accessible data due to study incompletion (11 participants). The remaining 136 participants’ data (n = 79 gynephilic, n = 57 androphilic) were used for analysis.

Participants were compensated eight dollars USD for the completion of the study.

#### Sample Size Justification

Our target sample size was 150 based on prior RL studies (Heller et al., 2018) and Study 1 (n = 137).

### Materials

We used the same set of fractal images as in Study 1.

All face photos came from the Chicago Face Database (CFD), which includes self-identified White, Black, Latino, and Asian male and female models recruited from the United States. We only included neutral facial expressions. Subjective rating norms of attractiveness were based on a U.S. rater sample in which participants rated the faces on a one to seven scale based on the following prompt: “Now, consider the person pictured above and rate him/her with respect to other people of the same race and gender” (Ma et al., 2015).

We selected 50 female faces with the highest attractiveness ratings (*M* = 4.73, *SD* = 0.23), 25 female faces that had medium attractiveness ratings (*M* = 3.43, *SD* = 0.08), and 50 female faces with the lowest attractiveness ratings (*M* = 2.26, *SD* = 0.28). Additionally, we selected 50 male faces with the highest attractiveness ratings (*M* = 3.99, *SD* = 0.35), 25 male faces that had medium attractiveness ratings (*M* = 3.18, *SD* = 0.04), and 50 male faces with the lowest attractiveness ratings (*M* = 2.14, *SD* = 0.22; Ma et al., 2015). Notably, the mean subjective attractiveness ratings for males were significantly lower than those of the females. We prioritized having the largest contrast possible between attractiveness levels within a gender rather than matching across genders. Therefore, across-gender results should be interpreted with this caveat in mind.

### Face-Rating Task

In the face-rating task, participants rated all 250 faces that they could possibly see in the Face-Choice task (125 female and 125 male faces) on their attractiveness from one (least attractive) to nine (most attractive).

### Face-Choice Task

The Face-Choice task was functionally identical to the Emotion-Choice task from Study 1 except for a couple of aspects. First, the two trial types consisted of viewing male faces and viewing female faces. Therefore, the advantageous and disadvantageous choices reflected probabilities of viewing faces varying in their attractiveness (high vs. medium vs. low). We also improved some logistical features such as increasing the time participants had to make a choice (from 1.5 to 2 seconds) to reduce missing trials and reducing the time of the intertrial interval to 2 seconds for better pacing. Participants’ were more likely (than chance: .5) to choose the advantageous option for female face trials (M = .53, CI_95_ [.51, .54], *t*(135) = 2.77, p = .006, but not for male face trials (M = .51, CI_95_ [.49, .53]), *t*(135) = 1.29, p = .198.

### Procedure

The procedure was also slightly different from Study 1. The study was still online – built with PsychoPy and uploaded to Pavlovia. This time, however, we recruited participants from online samples with Prolific instead of Introductory Psychology subject pool participants. After beginning the experiment, participants were directed to a Qualtrics survey to sign the consent form and fill out basic demographic information such as their age, gender, gender attraction preference, and race/ethnicity. Subsequently, the participants were directed to Pavlovia to engage in the face-rating and face-choice tasks described above. As in Study 1, participants were only told to make a choice and that their choice would be shown and that they would see a face afterwards. They were not informed of contingences between their choices and the faces, nor motivated to see the most attractive faces. The experiment took around 40 minutes to complete. Upon completion of the study, participants were redirected to Prolific to receive compensation.

### Analysis

#### Q-Learning kRL Model.

We used the same Q-Learning Models from Study 1 to estimate the cRVs of faces, except instead of separate k values for positive and negative, we estimated *κ*^H^ and *κ*^L^ to reflect cRV of more and less attractive faces, respectively (separately for male and for female faces). We again set a reference by parameterizing the starting value of medium attractiveness faces as the initial *Q* value. To be consistent with Study 1, we also estimated learning rate (*α*) and temperature factors (*β*).

#### Hierarchical Bayesian Analysis Using the Stan Package.

We used the same HBA as in Study 1 to estimate the parameters. We extracted the median parameters from the posterior distribution for input into subsequent statistics (see Table S2 for full reporting of parameters). We again report t-tests, 95% confidence intervals, and effect sizes.

#### Model testing.

As in Study 1, we again tested the cRV model’s out-of-sample bayesian prediction accuracy and showed that it was a better fit than the model using participants average explicit ratings as reward values for both the female face trials (cRV: elpd = −5204, SE = 191.1; ***αβExp***: elpd = −6199.1, SE = 130.9) and male face trials (cRV: elpd = −5499.7, SE = 200.7; ***αβExp***: elpd = −6851.8, SE = 93.1). Note, because of a coding error we did not have the individual rating for each trial so instead substituted the average explicit ratings for each outcome. We also calculated the predicted choices for each trial given participants’ estimated model parameters (in 100 iterations) and showed that these models predicted choice accuracy greater than chance (Female faces: *M* = .68, Wilcoxon’s test = 9143, *p* < .001; Male Faces: *M* = .66, Wilcoxon’s test = 8946, *p* < .001).

### Results

#### Explicit Face Attractiveness

To confirm that the faces were different in their explicit attractiveness and to explore whether attraction preference moderated this attractiveness, we conducted a 2 (Attraction Preference: gynephilic [male attracted] or androphilic [female attracted]) by 2 (Face Gender: female, male) × 3 (Face Attractiveness: high, medium or low) mixed ANOVA designs with Attraction Preference as the between-subjects factor, Face Gender and Face Attractiveness as the within-subjects factors (with greenhouse-geisser corrections for sphericity violations), and explicit attractiveness ratings as the dependent variable. We followed up interactions with the appropriate t-tests.

Confirming our manipulation check, there was a significant main effect of Face Attractiveness, *F*(1.21, 161.5) = 604.27, *p* <.001, η_p_^2^ = .818, in which participants rated the high attractive (normed) faces as being higher in attractiveness (*M* = 4.89, CI_95_ [4.66, 5.12]) than faces medium in attractiveness (*M* = 3.88, CI_95_ [3.67, 4.09]), *t*(134) = 23.13, *p* <.001, CI_95_ [3.69, 4.38], and medium attractiveness faces as being higher in attractiveness than faces lower in attractiveness (*M* = 2.86, CI_95_ [2.68, 3.04]), *t*(134) = 22.01, *p* <.001, CI_95_ [3.70, 4.43].

**Figure 5.**
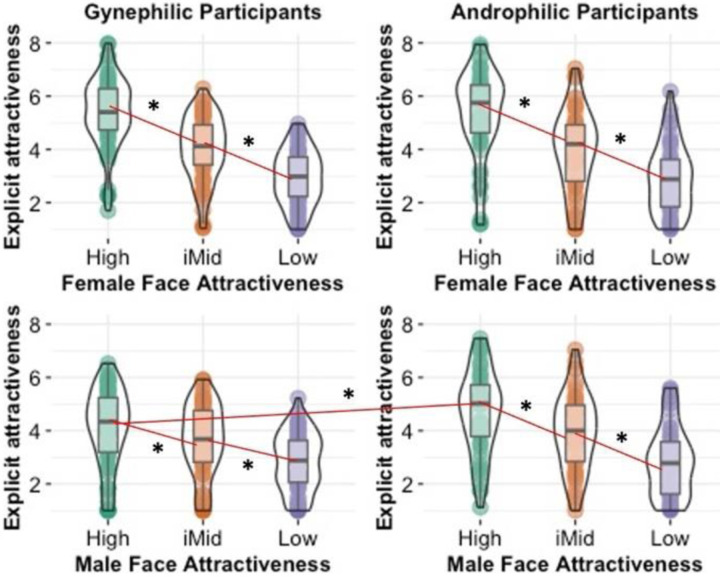
Study 2: Explicit attractiveness ratings as a function of attraction preference (gynephilic = attracted primarily to females; androphilic = attracted primarily to males) and gender of the face. * p < .05.

There were also a significant main effect of Face Gender, *F*(1,134) = 24.66, *p* <.001, η_p_^2^ = .155, and significant or marginally significant 2-way interactions of Attraction Preference and Face Attractiveness, *F*(1.21, 161.50) = 3.49, *p* =.056, η_p_^2^ = .025, and Face Attractiveness and Face Gender, *F*(1.72, 230.89) = 116.21, *p* <.001, η_p_^2^ = .464. These effects, however, were qualified by a significant 3-way interaction of Attraction Preference, Face Attractiveness, and Face Gender, *F*(1.72, 230.89) = 9.84, *p* <.001, η_p_^2^ = .068 (Figure 5). This interaction was due to a significant 2-way interaction of Attraction Preference and Face Attractiveness for male faces, *F*(1.28, 171.21) = 11.48, *p* <.001, η_p_^2^ = .079, whereas the interaction for female faces was not significant, *F*(1.30, 173.74) = .22, *p* =.705, η_p_^2^ = .002. The 2-way interaction for male faces was due to those participants attracted to males rating high attractive male faces as more attractive (*M* = 4.69, CI_95_ [4.30, 5.08]) than participants attracted to females (*M* = 4.11, CI_95_ [3.78, 4.44]), *t*(134) = 2.23, *p* =.028, CI_95_ [.06, 1.09], d = .39. Alternatively, the two attraction preference groups did not significantly differ in their ratings of medium attractive male faces (M_maleAtt_ = 3.92, CI_95_ [3.57, 4.28]; M_femAtt_ = 3.64, CI_95_ [3.33, 3.94]), *t*(134) = 1.21, *p* =.228, CI_95_ [-.18, .76], d = .21, and low attractive male faces (M_maleAtt_ = 2.82, CI_95_ [2.51, 3.12]; M_femAtt_ = 2.82, CI_95_ [2.57, 3.08]), *t*(134) = .03, *p* =.974, CI_95_ [-.41, .39], d = .006.

#### cRV for faces

To test our hypothesis that attractive faces would predict higher cRV than less attractive faces and to explore how this effect may be moderated by attraction preference and face gender, we conducted a 2 (Attraction Preference: gynephilic [male attracted] or androphilic [female attracted]) by 2 (Face Gender: female, male) × 2 (Face Attractiveness: high or low) mixed ANOVA designs with Attraction Preference as the between-subjects factor, Face Gender and Face Attractiveness as the within-subjects factors, and the k-values as the dependent variable. Because the k-values for each face gender category were separately estimated and initialized, we followed up interactions with t-tests and ANOVAs that compared effects within each face gender category.

**Figure 6.**
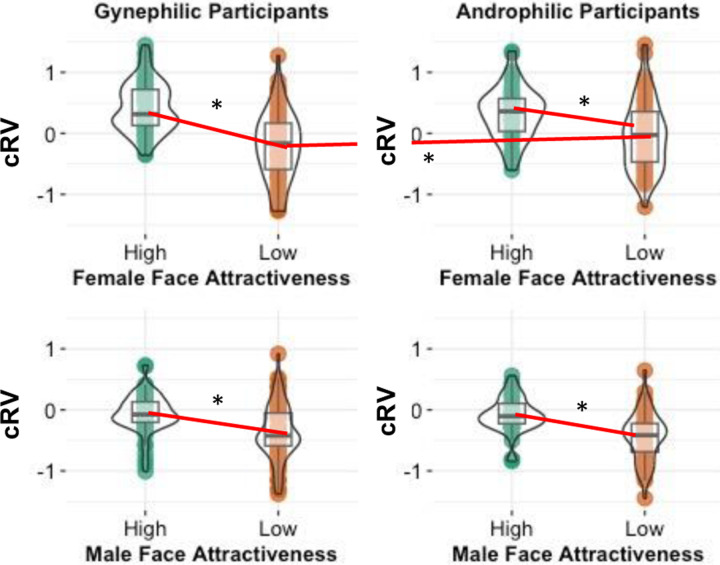
Study 2: computationally inferred reward values (cRV) as a function of attraction preference (gynephilic = attracted primarily to females; androphilic = attracted primarily to males) and gender of the face. * p < .05.

Supporting our primary hypothesis, there was a significant main effect of Face Attractiveness, *F*(1, 134) = 80.98, *p* <.001, η_p_^2^ = .377, in which cRV for high attractiveness faces was significantly higher (*M* = .16, CI_95_ [.11, .21]) than the CRV for low attractiveness faces (*M* = −.28, CI_95_ [-.35, −.21]). There were also a significant main effect of Face Gender, *F*(1,134) = 130.65, *p* <.001, η_p_^2^ = .494, and significant 2-way interactions of Attraction Preference and Face Gender, *F*(1, 134) = 4.09, *p* =.045, η_p_^2^ = .030, and Face Attractiveness and Face Gender, *F*(1, 134) = 8.21, *p* = .005, η_p_^2^ = .058. These effects, however, were qualified by a significant 3-way interaction of Attraction Preference, Face Attractiveness, and Face Gender, *F*(1, 134) = 5.59, *p* =.020, η_p_^2^ = .040 (Figure 6).

Interestingly, this interaction was the opposite as that found for the explicit ratings. It was due instead to a significant 2-way interaction of Attraction Preference and Face Attractiveness for female faces, *F*(1, 134) = 5.16, *p* =.025, η_p_^2^ = .037, whereas the interaction for male faces was not significant, *F*(1, 134) = .08, *p* =.780, η_p_^2^ < .001. The 2-way interaction for female faces was due to those participants attracted to females exhibiting lower cRVs for low attractiveness female faces (*M* = −.28, CI_95_ [−.41, −.14]) than participants attracted to males (*M* = −.03, CI_95_ [−.19, .12]), *t*(134) = 2.32, *p* =.022, CI_95_ [.04, .45], d = .40. Alternatively, the two attraction preference groups did not significantly differ in their cRVs of high attractive female faces (M_femAtt_ = .42, CI_95_ [.33, .52]); M_maleAtt_ = .35, CI_95_ [.24, .46]), *t*(134) = 0.96, *p* =.339, CI_95_ [−.22, .07], d = .17.

Another way to conceptualize this interaction is to compare the difference between high and low attractiveness faces as a function of face gender and attraction preference. Using contrast tests, the difference between cRV for high and low attractiveness female faces for those attracted to females was significantly higher than the cRV differences for the other three combinations (female faces:male attraction, male faces:female attraction, male faces:male attraction), *t*(134) = 3.52, *p* <.001, CI_95_ [.46 1.62], with no significant differences among the other three combinations, all *t*s < .6, *p*s > .95. Notably, these patterns were largely consistent with the 40-trial version except the 3-way interaction was not significant. Instead, the above cRV difference between high and low attractive female faces (compared to that for male faces) was found across attraction preferences (Supplementary Online Material).

#### Relationship between explicit and implicit

**Figure 7.**
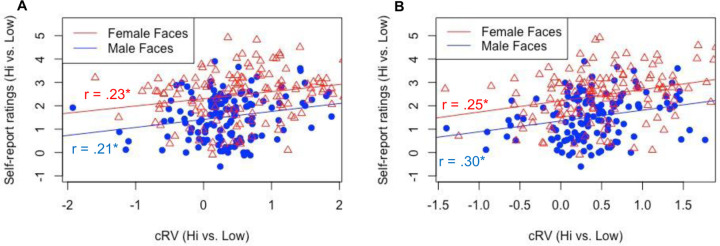
Study 2: Correlation between explicit ratings and computationally inferred reward values (cRV) for the 80 trial version (A) and 40 trial version (B). * p < .05

Lastly, as in Study 1, we explored the relationship between the explicit ratings and the cRVs within each face gender (Figure 7). When comparing each attractiveness level separately, the only significant correlation between explicit ratings and cRV was for low attractiveness female faces, *r*(134) = −.17, *p* =.044, with all other *r*s < |.11|, *p*s > .18. For the 40-trial version, none of these correlations were significant, all rs < |.16|. As in Study 1, because the cRVs are more interpretable when contrasted with each other, we computed cRV and explicit rating (high - low) difference scores and correlated these with each other. The explicit rating and cRV difference scores were mildly significantly positively correlated for both female faces, *r*(134) = .23, *p* =.008, and for male faces, r(134) = .21, *p* =.013 (Figure 7a). For the 40-trial version, these correlations were again significant (rs = .25, .30, for female and male faces, respectively, ps < .003; Figure 7b).

### Discussion

In Study 2, we demonstrated again that the kRL method was able to extract cRVs that follow the expected pattern, higher to viewing attractive faces than to viewing less attractive faces. Interestingly, the participants attracted primarily to females showed greater preference-congruent cRV sensitivity (high > low) than did the participants primarily attracted to males. This finding is consistent with findings that males (who were overwhelmingly attracted to females in our study) show greater gender-specificity in their physiological attraction than do females (Chivers, 2017). Like Study 1 (40-trial version), there was a positive correlation between explicit attractiveness ratings and cRVs, extending the validation of this method to assessing social reward values. However, this correlation was again small, the cRV models were better predictors of behavior than were the explicit ratings, and the overall explicit and cRV patterns were different, suggesting again that the cRVs may be assessing reward processes that are not necessarily fully captured by explicit value judgments.

## General discussion

In two studies, the kRL method successfully measured people’s reward values of their experiences. Although participants were not instructed on which categories of images they should try to view, their choices revealed that they were motivated to have experiences that were consistent with motivational reward theory (positive imagery, attractive faces). Their explicit value judgments also reflected this pattern, but these explicit values and cRVs were only mildly correlated. In addition, in both studies, calculating the cRVs led to better estimation of behavior than did using the explicit ratings as the reward values. These findings are consistent with theorizing and findings that as an indirect, non-self-report measure, cRV does not necessarily have to be tightly linked with explicit preferences (Anselme & Robinson, 2016). It is possible that there may have been some potential loss in translation when the participant’s implicit reward processes were made conscious and then reported, however, we cannot be certain of this formulation given that we did not measure explicit motivation throughout the task. Future research needs to explore this implicit-explicit relationship and the factors that may strengthen or weaken it (e.g., self-control; Fishbach & Shah, 2006).

This kRL method for measuring cRVs holds great promise for understanding a wide range of reward processes and individual differences. It can be used to measure cRVs to any category of stimuli because it is not limited to those high arousal stimuli that prime the musculature for action. For example, it could be used to investigate the relationship between depression and the associated changes in motivational reward values of stimuli and experiences that are not necessarily action-oriented (Sherdell et al., 2012; Treadway et al., 2012). Future studies should explore the relationship between this method and other methods for assessing implicit value processes to identify any points of convergence or divergence.

We also demonstrated that this method is sensitive to changes to cRVs, suggesting that it could be useful for assessing how they could be affected by time, drive state, and context. For example, it can be used to assess whether successful emotion regulation changes people’s cRV of emotional stimuli (Gyurak et al., 2011). Also, although we derived the cRVs from participants’ choice behavior, future investigations should assess whether these cRVs predict subsequent behavior. For example, it can be used in conjunction with the IAT to predict motivated behavior towards people of different races (Oswald et al., 2013).

In sum, despite the limitations of the present study, we have demonstrated that the kRL method for measuring cRV holds great potential for any field that cares about understanding one of the strongest causes of people’s motivated behavior.

### Constraints of generality statement

Our primary finding was that the kRL method seems to measure people’s cRVs toward various stimuli. Given that we showed this effect in both undergraduates and an online sample of adults, we have reason to believe that this general method would generalize to any population that is able to make a series of choices in a task. Although we showed this effect with pictorial stimuli, we believe that this effect would extend to any type of short-form experience that could be included in a series of short trials. To be seen is whether this effect also generalizes to more immersive, longer experiences. By the very nature of motivation we expect that the exact patterns of findings would necessarily be influenced by the reward state of the participants during the task including possibly dynamic changes in motivation throughout the task. We have no reason to believe that the results depend on other characteristics of the participants, materials, or context.

## Figures and Tables

**Table 1:** Model comparisons (Study 1)

Model	Elpd_loo	SE	Elpd_diff	SE_diff
***αβκ***	−6034.7	127.9	0	0
***αβExp***	−6037.9	128.5	−3.2	8.5
***αβ***	−6421.2	100.9	−386.4	82.9
***α***	−6509.2	86.2	−474.5	87.9

**Note.** Elpd: expected log pointwise predictive density; loo: leave one out; SE: standard error; diff: difference. The full model with computationally inferred reward parameters estimated ( ) provides better predictive density than models in which reward parameters are explicit (***Exp***), reward parameters are predetermined ( ) and just assessing learning rate alpha ( ).

## Data Availability

Study 1 was not preregistered; the preregistration for Study 2 can be accessed at https://osf.io/tak27. Deidentified data for both studies along with the data-analysis scripts are posted at https://osf.io/85wx3/. Researchers interested in assessing cRV for their own purposes can find a template for running this task and analyzing its data at https://gitlab.pavlovia.org/WakeEmoLab/rlposnegtemplate/.The materials used in these studies are widely available.
